# Exercise-induced hypertension, leukocyte telomere length, and dietary antioxidant intake in elite male taekwondo athletes: an integrated analysis

**DOI:** 10.3389/fphys.2026.1934571

**Published:** 2026-09-11

**Authors:** Yun-A Shin, Jun-Hee Lee

**Affiliations:** 1 Department of Exercise & Prescription, Dankook University, Cheonan-si, Republic of Korea; 2 Department of Sports Coaching, Kyunghee University, Yongin-si, Republic of Korea

**Keywords:** dietary fiber, exercise-induced hypertension, leukocyte telomere length, taekwondo athletes, vitamin C

## Abstract

**Purpose:**

This study examined leukocyte telomere length (LTL) and nutritional intake characteristics according to resting hypertension and exercise-induced hypertension (EIH) status exclusively in elite male collegiate and professional taekwondo athletes. It further investigated associations among blood pressure responses, biological aging markers, and nutritional indices without presuming a direct association between EIH and LTL.

**Methods:**

A total of 108 male taekwondo athletes underwent body composition assessment and graded exercise testing (GXT) using the Bruce treadmill protocol. Resting hypertension was defined as systolic blood pressure (SBP) ≥140 mmHg or diastolic blood pressure (DBP) ≥90 mmHg, and EIH as peak exercise SBP ≥210 mmHg or DBP ≥105 mmHg. Dietary intake was assessed via 3-day estimated food records and analyzed using CAN Pro 5.0. Relative LTL was measured by real-time PCR and expressed as the T/S ratio. Group comparisons employed Welch’s t-test; associations were examined using correlation, multiple linear regression, binary logistic regression, and generalized estimating equations.

**Results:**

Among 108 athletes, 23 (21.3%) met the resting hypertension criterion and 44 (40.7%) met the EIH criterion. The EIH group showed higher resting and peak SBP and a greater exercise-induced SBP rise. LTL did not differ between the EIH and non-EIH groups (p = .554) and was not independently associated with EIH odds (p = .177). LTL showed a borderline inverse association with resting SBP (ρ = −0.203, p = .050). In the multivariable models, BMI was inversely associated with LTL (B = −0.0070, p = .034) and positively associated with EIH odds (OR per 1-SD = 3.80, p = .002), whereas vitamin C intake density was inversely associated with EIH odds (OR per 1-SD = 0.30, p = .019). Dietary fiber and vitamin C intake densities were positively correlated with LTL (both p = .012).

**Conclusion:**

While EIH was not directly linked to telomere shortening in this cross-sectional observation, the high prevalence of EIH and its strong association with body composition and antioxidant intake highlight the necessity of dynamic blood pressure monitoring. Incorporating objective hydration controls and targeted nutritional strategies (e.g., vitamin C and fiber) should be considered as part of comprehensive cardiovascular risk management in elite athletes, warranting further prospective validation.

## 1 Introduction

Hypertension is a major risk factor for cardiovascular disease, cerebrovascular disease, and premature mortality, and is clinically defined as a resting systolic blood pressure (SBP) ≥140 mmHg or diastolic blood pressure (DBP) ≥90 mmHg (Lee et al., 2026). However, resting blood pressure measurements alone have inherent limitations, as hemodynamic responses during exercise more sensitively reflect vascular elasticity, peripheral resistance, cardiac afterload, and autonomic nervous system regulation (Nayor et al., 2023; da Silva et al., 2026). Although SBP physiologically increases during exercise in proportion to cardiac output, some individuals exhibit an exaggerated blood pressure rise at equivalent exercise intensities—a phenomenon termed exercise-induced hypertension (EIH) (Schultz and Sharman, 2014). EIH is generally defined as a peak exercise SBP ≥210 mmHg in males or ≥190 mmHg in females, and has garnered attention as a dynamic cardiovascular risk marker that predicts future hypertension, target organ damage, and cardiovascular mortality, even in individuals with normal resting blood pressure (Kosowski and Aleksandrowicz, 2025).

Cardiovascular risk assessment is critically important not only in the general population but also in elite athletes engaged in sustained high-intensity training (Franklin et al., 2020). Taekwondo training and competition involve repeated high-intensity efforts, and interval training can improve aerobic and anaerobic performance in combat-sport athletes (Franchini et al., 2019; Vasconcelos et al., 2020). owever, elevated peak exercise SBP in highly trained individuals may reflect physiological adaptation as well as potential cardiovascular risk, and its interpretation requires consideration of training status and exercise workload (Richard et al., 2021; Nayor et al., 2023). Therefore, characterizing stage-by-stage dynamic blood pressure responses and identifying EIH status are essential for the long-term cardiovascular health management of these athletes.

Telomere length, which protects chromosomal ends from degradation, is a pivotal biomarker of biological aging that shortens progressively with cell division and oxidative damage (Cawthon, 2002; Sack et al., 2017). Telomere attrition is known to be accelerated in hypertensive individuals through increased vascular wall tension, endothelial dysfunction, and low-grade systemic inflammation (Tellechea and Pirola, 2017). Compared to the well-documented relationship between resting hypertension and telomere length, however, evidence regarding the effects of exercise blood pressure responses or EIH on leukocyte telomere length (LTL) remains extremely limited. In elite taekwondo athletes, the generation of reactive oxygen species from intense training combined with the pronounced hemodynamic stress accompanying EIH may act as pathophysiological mechanisms that accelerate LTL shortening (Arsenis et al., 2017; Sack et al., 2017). Accordingly, elucidating the association between EIH and telomere length holds considerable scientific merit as a potential biomarker for predicting premature cardiovascular aging in athletes.

Furthermore, nutritional intake represents a critical modifiable lifestyle factor that may simultaneously influence both blood pressure and telomere length by modulating oxidative stress and vascular function (Galiè et al., 2020). Prior research has demonstrated that antioxidant nutrients, including dietary fiber and vitamin C, play a beneficial role in preserving telomere length and attenuating blood pressure elevation (Cai et al., 2023; Tucker, 2018). Rapid weight-loss practices before competition, including restricted food and fluid intake, have been reported in judo and taekwondo athletes (Berkovich et al., 2019). Rapid pre-competition weight reduction may also be accompanied by changes in arterial stiffness and hydration status (Koshiba and Maeshima, 2026), although its relationships with EIH and telomere length remain to be established. Nevertheless, no study to date has comprehensively examined resting blood pressure, EIH, telomere length, and nutritional status in an integrated framework among competitive athletes.

Therefore, this study aimed to compare LTL and nutritional intake characteristics according to resting hypertension and EIH status in elite taekwondo athletes using graded exercise testing. Specifically, we sought to investigate the interrelationships among blood pressure response indices (resting and peak exercise SBP), biological aging markers (telomere length), and key dietary variables (dietary fiber, vitamin C, protein, and sodium-potassium balance). The findings of this study are expected to underscore the clinical importance of monitoring both resting and exercise blood pressure in elite athletes, and to provide a scientific basis for developing sport nutrition intervention strategies aimed at preventing premature cardiovascular aging.

## 2 Materials and methods

### 2.1 Participants

This study enrolled 108 male elite taekwondo athletes in their twenties, comprising collegiate athletes registered with the Korea University Sport Federation (KUSF) and professional athletes affiliated with provincial sports councils in the Gyeonggi and Chungcheongnam-do regions. Participants were recruited between December 2023 and December 2024 via an online website, campus bulletin boards, and coordination with regional sports councils. For the prespecified dichotomous analyses, 85 athletes were classified as Non-hypertensive and 23 as having resting hypertension; 64 were classified as non-EIH and 44 as EIH in the full cohort. Complete-case EIH group comparisons included 58 non-EIH and 40 EIH athletes. Prior to participation, all athletes received a detailed explanation of the study objectives, procedures, and potential risks, after which written informed consent was obtained. The study was reviewed and approved by the Institutional Review Board of Dankook University (Approval No.: DKU-2021-11-006).

### 2.2 Measurements

#### 2.2.1 Body composition

Height (cm) was measured using an automated stadiometer. Body mass (kg), body mass index (BMI, kg/m²), body fat percentage (%), and skeletal muscle mass (kg) were assessed before GXT using a multi-frequency bioelectrical impedance analysis device (InBody 770, Biospace Co., Ltd., Seoul, Korea). Waist circumference (cm) was measured at the midpoint between the inferior margin of the last rib and the iliac crest while the participant stood erect, at the end of a normal expiration. Urine specific gravity, urine osmolality, or plasma osmolality was not measured, and no formal euhydration criterion was applied. Therefore, the potential influence of hydration status on BIA-derived body-composition estimates could not be excluded.

#### 2.2.2 Resting blood pressure measurement and classification

Resting blood pressure (BP) was measured after participants had been seated quietly for a minimum of 5 minutes following arrival at the laboratory. An appropriately sized cuff was positioned over the brachial artery at heart level, and BP was measured using an automated sphygmomanometer (Tango+, SunTech Medical, Morrisville, NC, USA) in accordance with American College of Sports Medicine guidelines. A minimum of two measurements were obtained at intervals of at least 1 minute, and the average was used for analysis. Consistent with the 2026 Korean Society of Hypertension guidelines (Lee et al., 2026), participants with SBP <140 mmHg and DBP <90 mmHg were classified as Non-hypertensive, whereas those with SBP ≥140 mmHg and/or DBP ≥90 mmHg were classified as having resting hypertension. Thus, values of SBP 120–139 mmHg and/or DBP 80–89 mmHg were included in the non-hypertensive category in this dichotomous analysis.

#### 2.2.3 Graded exercise testing and classification of exercise-induced hypertension

Graded exercise testing (GXT) was conducted on a motorized treadmill using the standard Bruce protocol to assess cardiorespiratory fitness and dynamic blood pressure responses. The Bruce protocol was selected because it provides a standardized maximal treadmill test with simultaneous respiratory gas and stage-specific BP assessment; however, its 3-minute stages and relatively large workload increments limit fine-grained characterization of BP transitions between adjacent exercise intensities. Respiratory gas exchange was analyzed using the Quark CPET system (Cosmed, Rome, Italy), and heart rate (HR) was continuously monitored using a Polar H7 monitor (Polar Electro, Kempele, Finland). BP at each exercise stage and at peak exercise was measured using a stress-testing sphygmomanometer (Tango+, SunTech Medical, Morrisville, NC, USA). Perceived exertion was assessed using Borg’s 6–20 scale (RPE). Test termination criteria were met when two or more of the following were satisfied: (i) respiratory exchange ratio (RER) ≥1.10; (ii) VO_2_ or HR plateau despite increasing intensity; (iii) RPE ≥17; or (iv) volitional fatigue. Forced termination followed ACSM guidelines (SBP >250 mmHg or DBP >115 mmHg). EIH was defined as peak exercise SBP ≥210 mmHg and/or DBP ≥105 mmHg during GXT (Richard et al., 2021).

#### 2.2.4 Dietary assessment

Dietary intake was assessed using a 3-day estimated food record (two weekdays and one weekend day) completed contemporaneously with the laboratory assessment during the athletes’ ongoing training period. Participants received standardized guidance from trained graduate research assistants, and follow-up interviews were conducted to supplement missing data. Visual aids were provided to improve portion-size estimation. Dietary data were analyzed using CAN Pro 5.0 (Korean Nutrition Society) and compared with the Korean Dietary Reference Intakes. Proximity to competition and whether participants were actively undertaking rapid weight loss were not prospectively recorded; therefore, these records cannot be interpreted as representing a standardized off-season or pre-competition dietary phase.

#### 2.2.5 Leukocyte telomere length analysis

Ten milliliters of peripheral blood were collected into EDTA tubes. Genomic DNA was extracted using the QIAamp DNA Blood Mini Kit (Qiagen, Hilden, Germany). Relative LTL was measured by real-time qPCR (QuantStudio™ 5, Thermo Fisher Scientific, Waltham, MA, USA) following the method of Cawthon (5). The telomere-to-single-copy gene (T/S) ratio relative to the reference gene *36B4* was used as an index of telomere length. PCR conditions: initial denaturation at 94 °C for 3min, then 35 cycles of 94 °C/30s, 56 °C/25s, 72 °C/30s, with a final extension at 72 °C for 7min. Amplification specificity was verified by melt curve and standard curve analyses.

### 2.3 Statistical analysis

All analyses were performed using IBM SPSS Statistics (v26.0; IBM Corp., Armonk, NY, USA). Descriptive statistics are expressed as mean ± SD. Distributional assumptions were evaluated using the Shapiro–Wilk test and visual inspection of Q–Q plots, and homogeneity of variance was assessed using Levene’s test. Because group sizes were unequal and several outcomes showed unequal variances, between-group comparisons were conducted using Welch’s independent t-test. Effect sizes were reported as Cohen’s d (small: 0.2; medium: 0.5; large: 0.8). Spearman rank correlations were used for associations involving variables that did not satisfy parametric distributional assumptions; Pearson correlations were reserved for approximately normally distributed continuous variables. Independent predictors of LTL were examined using multiple linear regression, with multicollinearity assessed using the variance inflation factor (VIF). EIH correlates were examined using binary logistic regression, reporting odds ratios (ORs) and 95% confidence intervals (CIs); model fit was assessed using the likelihood-ratio test and pseudo-R². Stage-by-stage BP trajectories were analyzed using generalized estimating equations (GEE) with an exchangeable correlation structure. All statistical tests were two-tailed with α = .05.

## 3 Results

### 3.1 General characteristics of participants

A total of 108 elite male taekwondo athletes were included. Resting hypertension was identified in 23 athletes (21.3%), and EIH was identified in 44 athletes (40.7%) in the full cohort. Because of incomplete data for selected variables(missing dietary records [n=8] and inadequate blood samples for LTL analysis [n=2]), complete-case sample sizes varied across analyses; the EIH group comparison in **Table 1** included 98 athletes. Detailed characteristics are presented in **Table 2**.

**Table 1:** Comparison of characteristics according to exercise-induced hypertension (EIH) status.

Variable	Non-EIH (n=58)	EIH (n=40)	p	Cohen’s d
Age, y	26.24 ± 4.23	26.90 ± 3.77	.421	0.16
BMI, kg/m²	20.74 ± 3.58	22.03 ± 2.93	.052	0.39
Body fat, %	16.39 ± 8.65	12.53 ± 4.89	.006**	−0.53
Telomere length, T/S	1.319 ± 0.060	1.310 ± 0.079	.554	−0.13
VO_2_max, mL/kg/min	53.07 ± 12.33	56.33 ± 10.25	.181	0.28
Resting SBP, mmHg	120.77 ± 11.76	127.55 ± 11.83	.007**	0.58
Resting DBP, mmHg	76.00 ± 12.49	76.18 ± 16.48	.955	0.01
Peak exercise SBP, mmHg	182.86 ± 17.04	226.62 ± 10.61	<.001**	2.96
ΔSBP, mmHg	62.89 ± 17.10	99.08 ± 11.74	<.001**	2.39
Energy intake, kcal/day	1333.63 ± 427.16	1504.79 ± 528.27	.092	0.36
Protein, g/kg/day	0.97 ± 0.37	0.94 ± 0.38	.715	−0.08
Dietary fiber, g/1000 kcal	7.89 ± 1.93	7.54 ± 2.23	.420	−0.17
Vitamin C, mg/1000 kcal	27.80 ± 12.60	22.37 ± 10.99	.025*	−0.45
Na/K ratio	1.94 ± 0.64	1.83 ± 0.49	.494	−0.18

Values are mean ± SD. Complete-case analysis included 98 athletes (58 non-EIH and 40 EIH); EIH status was available for all 108 participants. EIH was defined as peak exercise SBP ≥210 mmHg and/or DBP ≥105 mmHg. p-values are from Welch’s independent t-test. Cohen’s d: positive values indicate higher values in the EIH group. *p <.05; **p <.01.

**Table 2:** General characteristics of the participants (n=108).

Variable	Mean ± SD or n (%)	Range
Age, y	25.66 ± 4.19	21.00–29.00
Height, cm	169.57 ± 11.53	140.00–200.00
Weight, kg	62.24 ± 15.17	31.80–102.00
BMI, kg/m²	21.36 ± 3.37	15.12–30.35
Body fat, %	14.92 ± 7.53	3.40–37.00
Fat-free mass, kg	29.60 ± 7.68	13.40–49.50
Fat mass, kg	9.60 ± 5.78	1.40–27.50
Telomere length, T/S ratio	1.315 ± 0.068	0.930–1.439
VO_2_max, mL/kg/min	54.38 ± 11.50	13.50–74.60
Resting SBP, mmHg	123.53 ± 12.14	95.00–155.00
Resting DBP, mmHg	76.06 ± 14.14	42.00–108.00
Peak exercise SBP, mmHg	200.55 ± 26.12	147.00–251.00
Peak exercise DBP, mmHg	80.11 ± 14.26	43.00–126.00
ΔSBP, mmHg	77.81 ± 23.39	24.00–123.00
Energy intake, kcal/day	1417.20 ± 502.52	476.17–2877.88
Protein, g/kg/day	0.96 ± 0.37	0.41–2.35
Dietary fiber, g/1000 kcal	7.72 ± 2.07	3.02–16.83
Vitamin C, mg/1000 kcal	25.71 ± 12.45	0.49–58.75
Sodium, mg/1000 kcal	2016.02 ± 595.54	636.26–3969.42
Potassium, mg/1000 kcal	1094.94 ± 294.77	533.16–2402.69
Na/K ratio	1.90 ± 0.56	0.60–4.34
Resting hypertension, n (%)	23 (21.3%)	—
Exercise-induced hypertension, n (%)	44 (40.7%)	—

Values are presented as mean ± SD or n (%). BMI, body mass index; SBP, systolic blood pressure; DBP, diastolic blood pressure; ΔSBP, peak exercise SBP − resting SBP. Resting hypertension was defined as SBP ≥140 mmHg and/or DBP ≥90 mmHg. EIH was defined as peak exercise SBP ≥210 mmHg and/or DBP ≥105 mmHg.

### 3.2 Between-group comparisons according to resting hypertension status

Between-group comparisons revealed significant differences in cardiovascular indices (**Table 3**). The hypertensive group demonstrated significantly higher resting SBP (p=.003, d=1.00) and DBP (p<.001, d=1.46) and peak exercise SBP (p=.011, d=0.70) relative to the Non-hypertensive group. The ΔSBP tended to be greater in the hypertensive group but did not reach significance (p=.308). LTL showed a non-significant declining trend in the hypertensive group (p=.214). No significant differences were found for age, BMI, body fat, VO_2_max, or dietary variables.

**Table 3:** Comparison of characteristics according to resting hypertension status.

Variable	Non-hypertensive (n=85)	Resting hypertension (n=23)	p	Cohen’s d
Age, y	26.07 ± 4.09	27.52 ± 3.42	.097	0.37
BMI, kg/m²	21.14 ± 3.53	21.58 ± 2.88	.552	0.13
Body fat, %	14.89 ± 7.87	13.57 ± 6.03	.400	−0.18
Telomere length, T/S	1.320 ± 0.068	1.299 ± 0.068	.214	−0.31
VO_2_max, mL/kg/min	54.45 ± 11.46	54.15 ± 11.90	.920	−0.03
Resting SBP, mmHg	120.89 ± 9.55	132.13 ± 15.54	.003**	1.00
Resting DBP, mmHg	71.94 ± 11.56	89.55 ± 13.60	<.001**	1.46
Peak exercise SBP, mmHg	197.27 ± 23.74	214.61 ± 27.89	.011*	0.70
ΔSBP, mmHg	76.36 ± 22.74	82.48 ± 25.35	.308	0.26
Energy intake, kcal/day	1371.93 ± 482.04	1529.40 ± 474.63	.174	0.33
Protein, g/kg/day	0.97 ± 0.38	0.92 ± 0.33	.547	−0.13
Dietary fiber, g/1000 kcal	7.71 ± 2.19	7.86 ± 1.60	.733	0.07
Vitamin C, mg/1000 kcal	25.89 ± 12.80	24.76 ± 10.47	.671	−0.09
Na/K ratio	1.86 ± 0.63	2.06 ± 0.28	.125	0.35

Values are mean ± SD. p-values are from Welch’s independent t-test. Cohen’s d: positive values indicate higher values in the resting hypertension group. Resting hypertension was defined as SBP ≥140 mmHg and/or DBP ≥90 mmHg. *p <.05; **p <.01. SBP, systolic blood pressure; DBP, diastolic blood pressure; ΔSBP, exercise-induced SBP rise.

The mean SBP and DBP values in the resting hypertension group appear lower than the ≥140/90 mmHg threshold due to the combined inclusion of athletes with isolated systolic hypertension and those with isolated diastolic hypertension.

### 3.3 Between-group comparisons according to exercise-induced hypertension status

Between-group comparisons according to EIH status revealed markedly elevated hemodynamic responses and lower vitamin C intake in the EIH group (**Table 1**). Resting SBP was significantly higher in the EIH group (p=.007, d=0.58), whereas resting DBP did not differ (p=.955). Peak exercise SBP (p<.001, d=2.96) and ΔSBP (p<.001, d=2.39) were markedly elevated in the EIH group. Body fat percentage was significantly lower in the EIH group (p=.006, d=−0.53), whereas BMI showed a borderline tendency to be higher (p=.052). LTL did not differ between groups (p=.554). Vitamin C intake density was significantly lower in the EIH group (p=.025, d=−0.45).

### 3.4 Stage-by-stage blood pressure responses during graded exercise testing

**Figure 1:**
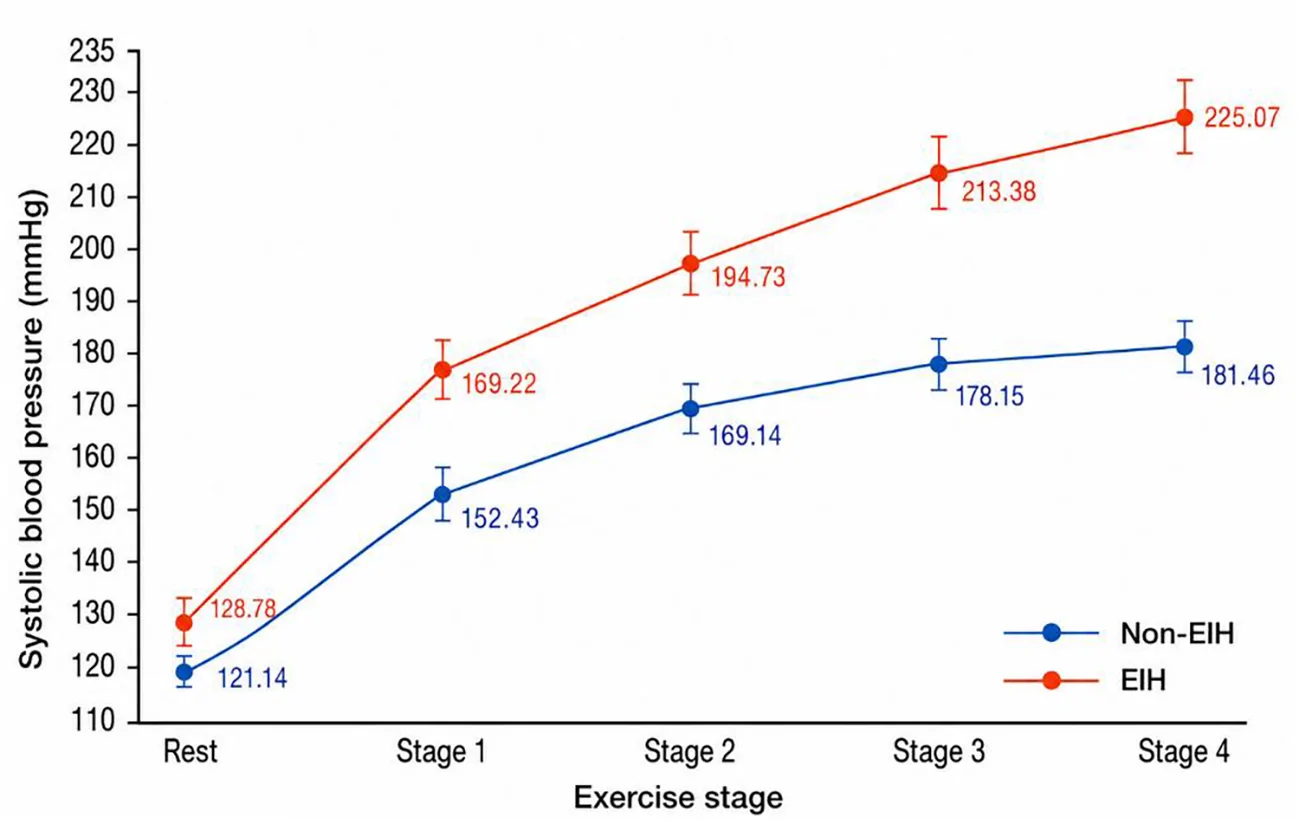
Changes in systolic blood pressure across exercise stages in the non-EIH and EIH groups. Values are mean ± SE. Stage 5 omitted because few participants reached this stage. EIH, exercise-induced hypertension.

**Figure 2:**
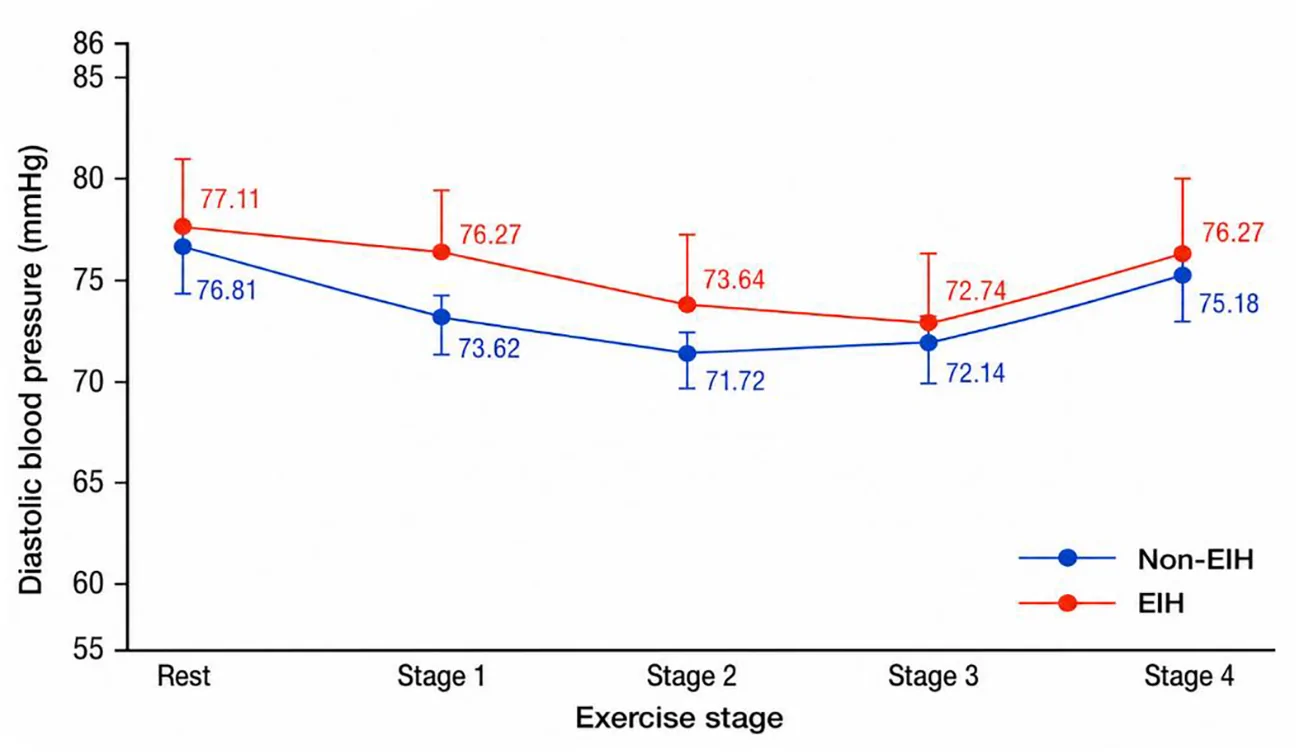
Changes in diastolic blood pressure across exercise stages in the non-EIH and EIH groups. Values are mean ± SE. Stage 5 omitted because few participants reached this stage. EIH, exercise-induced hypertension.

SBP trajectories across exercise stages differed significantly by EIH status (**Figures 1**, **2**). GEE analysis revealed significant main effects of group (Wald χ²=11.38, df=1, p=.0007), stage (Wald χ²=974.41, df=4, p<.001), and a significant group×stage interaction (Wald χ²=109.26, df=4, p<.001). *Post hoc* testing confirmed that the EIH group maintained significantly higher SBP at every measurement point from rest (EIH: 128.78mmHg vs. Non-EIH: 121.14mmHg) through Stage 4 (EIH: 225.07mmHg vs. Non-EIH: 181.46mmHg) (all p<.001). For DBP, only a significant stage effect was observed (Wald χ²=10.26, df=4, p=.036); the group effect (p=.652) and group×stage interaction (p=.873) were not significant.

### 3.5 Correlations between telomere length and cardiometabolic or nutritional variables

Spearman correlation results are presented in **Table 4**. LTL showed a borderline-significant negative association with resting SBP (ρ=−0.203, p=.050). Dietary fiber intake density (ρ=0.258, p=.012) and vitamin C intake density (ρ=0.256, p=.012) were significantly and positively correlated with LTL. No other variables reached statistical significance.

**Table 4:** Spearman correlations between telomere length and cardiometabolic or nutritional variables.

Variable	ρ	p
Age, y	−0.115	.267
BMI, kg/m²	−0.078	.455
VO_2_max, mL/kg/min	−0.083	.448
Resting SBP, mmHg	−0.203	.050†
Resting DBP, mmHg	−0.132	.216
Peak exercise SBP, mmHg	−0.097	.353
ΔSBP, mmHg	−0.027	.799
Energy intake, kcal/day	0.115	.266
Protein, g/kg/day	0.170	.099
Dietary fiber, g/1000 kcal	0.258	.012*
Vitamin C, mg/1000 kcal	0.256	.012*
Sodium, mg/1000 kcal	0.138	.333
Potassium, mg/1000 kcal	0.157	.270
Na/K ratio	0.027	.851
Calcium, mg/1000 kcal	0.146	.157

ρ, Spearman’s rank correlation coefficient. Telomere length expressed as T/S ratio. Energy-adjusted nutrient variables calculated per 1000kcal. †borderline significance (p=.050); *p<.05.

### 3.6 Regression analyses for telomere length and exercise-induced hypertension risk

Results of Model A (multiple linear regression for LTL) and Model B (logistic regression for EIH) are presented in **Table 5**.

**Table 5:** Multivariable models for telomere length (Model A) and exercise-induced hypertension risk (Model B).

Model A. Multiple linear regression for telomere length (T/S ratio)					
Predictor	B	SE		β	p
Age	0.0033	0.0028		0.182	0.245
BMI	−0.0070	0.0032		−0.362	.034*
VO_2_max	−0.0001	0.0007		−0.009	0.94
Resting SBP	−0.0007	0.0006		−0.134	0.227
EIH status	0.019	0.0154		0.146	0.219
Protein, g/kg/day	0.0211	0.0213		0.123	0.325
Dietary fiber, g/1000 kcal	0.0065	0.0051		0.208	0.211
Vitamin C, mg/1000 kcal	0.0007	0.0008		0.123	0.44
Model B. Binary logistic regression for exercise-induced hypertension					
Predictor	OR (per 1-SD)		95% CI		p
Age	0.49		0.23–1.04		0.063
BMI	3.8		1.64–8.85		.002**
VO_2_max	1.73		0.97–3.07		0.063
Resting SBP	1.7		0.98–2.96		0.06
Telomere length (T/S)	1.45		0.84–2.51		0.177
Protein, g/kg/day	1.41		0.76–2.64		0.277
Dietary fiber, g/1000 kcal	1.94		0.78–4.83		0.157
Vitamin C, mg/1000 kcal	0.3		0.11–0.82		.019*

Model A: n=108, R²=.176, adjusted R²=.089, model p=.054. Model B: n=108, pseudo-R²=.200, likelihood-ratio p=.003. In Model B, continuous predictors were standardized; OR indicates change in odds per 1-SD increase. BMI, body mass index; SBP, systolic blood pressure; EIH, exercise-induced hypertension; OR, odds ratio; CI, confidence interval. *p<.05; **p<.01.

Model A — Predictors of telomere length: The model explained 17.6% of LTL variance (R²=.176; adjusted R²=.089; p=.054). BMI was the only significant independent negative predictor (B=−0.0070, β=−0.362, p=.034). No other predictor reached significance.

Model B — Predictors of EIH risk: The logistic model was significant (p=.003; pseudo-R²=.200). Each 1-SD increase in BMI raised EIH risk 3.80-fold (OR=3.80, 95% CI: 1.64–8.85, p=.002), whereas each 1-SD increase in vitamin C intake density reduced EIH risk by 70% (OR=0.30, 95% CI: 0.11–0.82, p=.019). LTL was not associated with EIH risk (p=.177).

## 4 Discussion

This study investigated the interrelationships among resting BP, exercise BP responses, LTL, and nutritional intake in elite male taekwondo athletes. The EIH group exhibited higher resting and peak SBP and a greater SBP rise than the non-EIH group. Importantly, LTL neither differed by EIH status nor independently predicted EIH odds, indicating that the exercise BP phenotype observed here was not accompanied by detectable differences in this cumulative biological-aging marker. LTL showed a borderline inverse association with resting SBP and was inversely associated with BMI in the multivariable model. Dietary fiber and vitamin C intake densities correlated positively with LTL, and vitamin C intake density was inversely associated with EIH odds. These cross-sectional findings identify potentially relevant correlates but do not establish causal or protective effects.

### 4.1 Hemodynamic characteristics of EIH in elite taekwondo athletes

EIH prevalence of 40.7% observed in this cohort substantially exceeds the reported general population prevalence and underscores the significant cardiovascular burden present even in a physically elite population. These findings are consistent with prior evidence indicating that EIH reflects not merely an exaggerated physiological response to increasing exercise intensity, but rather a sensitive indicator of reduced vascular elasticity, increased arterial stiffness, elevated cardiac afterload, and dysregulated autonomic nervous system modulation — even in individuals with normal resting blood pressure (Schultz and Sharman, 2014; Kosowski and Aleksandrowicz, 2025). Exaggerated BP elevation during exercise is widely regarded as a robust dynamic predictor of left ventricular hypertrophy, target organ damage, and future hypertension risk (Schultz and Sharman, 2014; Zafrir et al., 2022).

Notably, DBP responses did not differ between EIH and non-EIH groups at any exercise stage — a finding consistent with the typical hemodynamic adaptation observed in athletes, whereby DBP is maintained or modestly reduced during exercise due to increased skeletal muscle blood flow and peripheral vasodilation (Schultz and Sharman, 2014). The stage-by-stage GEE analysis further confirmed a significant group and stage interaction for SBP (Wald χ² = 109.26, p <.001), with the EIH group maintaining consistently elevated SBP from rest through peak exercise, whereas no such group-by-stage divergence was evident for DBP. This selective SBP exaggeration without DBP elevation is characteristic of a sympathetically driven, high-cardiac-output hemodynamic phenotype (Cseh et al., 2025; Romano et al., 2026), and warrants careful monitoring in athlete populations.

### 4.2 Body composition and EIH risk: beyond the conventional BMI interpretation

A noteworthy finding was the divergence in body-composition estimates within the EIH group. Although the EIH group tended to have a higher BMI (p = .052), its BIA-derived body fat percentage was lower (p = .006, d = −0.53). This pattern may be compatible with greater lean mass in trained athletes, but it should not be interpreted as definitive evidence of greater muscularity because hydration status was not objectively verified and BIA estimates are hydration-sensitive. The logistic regression model indicated that each 1-SD increase in BMI was associated with 3.80-fold higher odds of EIH (OR = 3.80, 95% CI: 1.64–8.85, p = .002). Potential explanations include higher cardiac output and peripheral vascular compression during maximal exertion in athletes with greater active muscle mass (Mitchell et al., 1981), but direct measures of lean mass, fluid status, and vascular function are needed. Elevated BMI in combat-sport athletes should therefore be interpreted using multidimensional assessment rather than a conventional obesity lens alone.

### 4.3 Telomere length, resting blood pressure, and the temporal dissociation from EIH

LTL showed a borderline-significant negative association with resting SBP (ρ = −0.203, p = .050), consistent with the established pathophysiological cascade in which hypertension-related increases in vascular wall tension, low-grade systemic inflammation, and oxidative stress collectively accelerate telomere attrition and cellular senescence (Baek et al., 2025; Tellechea and Pirola, 2017). However, LTL did not differ significantly between EIH and non-EIH groups (p = .554), nor was EIH a significant predictor of LTL in the regression model (p = .177).

This apparent dissociation is mechanistically plausible: LTL reflects cumulative, years-long metabolic burden and lifestyle exposures (Baek et al., 2025), whereas peak exercise SBP is primarily determined by acute sympathetic activation, cardiac output, and peripheral vascular reactivity on the day of testing (Houben et al., 2008; Schultz and Sharman, 2014). In athletes without overt cardiovascular disease, transient exercise-induced BP elevations may represent physiological adaptation rather than fixed vascular pathology (Richard et al., 2021). Exercise intensity and training status also influence BP responses: young trained individuals exhibited smaller SBP increases and faster recovery after a Master two-step test than untrained individuals (Nakamura et al., 2021). Accordingly, EIH in taekwondo athletes should be interpreted as a dynamic cardiovascular risk signal rather than a surrogate for established vascular aging. Longitudinal studies tracking LTL attrition rates and hypertension conversion in EIH-positive athletes are needed to clarify the long-term significance of this phenotype.

### 4.4 Dietary antioxidant nutrients as modulators of telomere length and EIH risk

Dietary intake variables emerged as important bridging factors linking blood pressure regulation and biological aging in this study. Both dietary fiber density (ρ = 0.258, p = .012) and vitamin C intake density (ρ = 0.256, p = .012) were significantly and positively correlated with LTL, supporting the view that dietary patterns rich in plant-based antioxidants and anti-inflammatory nutrients attenuate oxidative DNA damage and thereby preserve telomere integrity (Cai et al., 2023; Tucker, 2018).

Vitamin C intake density was lower in the EIH group than in the non-EIH group (p = .025, d = −0.45), and a 1-SD higher vitamin C intake density was associated with 70% lower odds of EIH in the logistic model (OR = 0.30, 95% CI: 0.11–0.82, p = .019). A plausible mechanism involves reduced endothelial oxidative stress and greater nitric oxide bioavailability (Cai et al., 2023). Nevertheless, the cross-sectional design, short dietary recording period, and unmeasured competition or weight-cutting phase preclude causal interpretation. Thus, vitamin C and dietary fiber density should be considered candidate nutritional correlates for prospective testing rather than proven interventions for reducing exercise BP or slowing cellular aging.

Sodium, potassium, and the Na/K ratio were not significantly associated with LTL or EIH status. The 3-day food record has limited ability to characterize habitual long-term sodium intake (McLean et al., 2018), and the athletes’ competition proximity and active weight-loss status were not recorded. Consequently, short-term dietary variation related to training periodization or weight making may have introduced exposure misclassification and attenuated associations with BP outcomes.

### 4.5 Methodological considerations and generalizability

This study has several methodological limitations. First, the standard Bruce protocol contains relatively large workload increments; therefore, the stage-by-stage results describe responses at discrete stages but cannot resolve fine-grained BP transitions across small intensity increments. Second, hydration was not objectively verified before BIA, so the lower body-fat estimate in the EIH group may partly reflect fluid-status variation. Third, the 3-day food record was not anchored to a standardized phase of the competitive calendar, and active weight-cutting status was not documented. Finally, the cross-sectional design, male-only sample, and single-sport recruitment preclude causal inference.

The comprehensive assessment battery may make these findings relevant to other young, highly trained male athletes, particularly those in combat or weight-class sports who experience repeated high-intensity hemodynamic stress. However, generalization to female athletes, older athletes, endurance athletes, recreationally active adults, or clinical populations requires caution because sex, training phenotype, vascular adaptation, and weight-management practices may modify exercise BP and LTL relationships.

## 5 Conclusion

In conclusion, EIH was common among elite male taekwondo athletes, but EIH was neither associated with LTL in group comparisons nor an independent correlate of LTL in multivariable analysis. Higher BMI and lower vitamin C intake density were associated with EIH odds, whereas dietary fiber and vitamin C intake densities were positively correlated with LTL. These associations are hypothesis-generating and should not be interpreted as evidence that antioxidant intake prevents EIH or telomere attrition. Longitudinal studies that include female athletes, objective hydration markers, repeated dietary assessments across training phases, and protocols with smaller workload increments are needed before broader clinical or sport-nutrition recommendations can be made.

## Data Availability

The datasets presented in this article are not readily available because of privacy and ethical restrictions. Requests to access the datasets should be directed to shinagel3@gmail.com.
